# Unsupervised machine learning identifies biomarkers of disease progression in post-kala-azar dermal leishmaniasis in Sudan

**DOI:** 10.1371/journal.pntd.0012924

**Published:** 2025-03-11

**Authors:** Ana Torres, Brima Musa Younis, Samuel Tesema, Jose Carlos Solana, Javier Moreno, Antonio J. Martín-Galiano, Ahmed Mudawi Musa, Fabiana Alves, Eugenia Carrillo

**Affiliations:** 1 WHO Collaborating Centre for Leishmaniasis. Spanish National Centre for Microbiology, Instituto de Salud Carlos III, Majadahonda (Madrid), Spain; 2 CIBER de Enfermedades Infecciosas, Instituto de Salud Carlos III, Madrid, Spain; 3 Escuela de Doctorado, Universidad Autónoma de Madrid, Madrid, Spain; 4 Institute of Endemic Diseases, University of Khartoum, Khartoum, Sudan; 5 Drugs for Neglected Diseases Initiative, Nairobi, Kenya; 6 Core Scientific and Technical Units, Instituto de Salud Carlos III, Madrid, Spain; 7 Drugs for Neglected Diseases Initiative, Geneva, Switzerland; University of Antwerp Drie Eiken Campus: Universiteit Antwerpen Campus Drie Eiken, BELGIUM

## Abstract

**Background:**

Post-kala-azar dermal leishmaniasis (PKDL) appears as a rash in some individuals who have recovered from visceral leishmaniasis caused by *Leishmania donovani*. Today, basic knowledge of this neglected disease and how to predict its progression remain largely unknown.

**Methods and findings:**

This study addresses the use of several biochemical, haematological and immunological variables, independently or through unsupervised machine learning (ML), to predict PKDL progression risk. In 110 patients from Sudan, 31 such factors were assessed in relation to PKDL disease state at the time of diagnosis: progressive (worsening) versus stable. To identify key factors associated with PKDL worsening, we used both a conventional statistical approach and multivariate analysis through unsupervised ML. The independent use of these variables had limited power to predict skin lesion severity in a baseline examination. In contrast, the unsupervised ML approach identified a set of 10 non-redundant variables that was linked to a 3.1 times higher risk of developing progressive PKDL. Three of these clustering factors (low albumin level, low haematocrit and low IFN-γ production in PBMCs after *Leishmania* antigen stimulation) were remarkable in patients with progressive disease. Dimensionality re-establishment identified 11 further significantly modified factors that are also important to understand the worsening phenotype. Our results indicate that the combination of anaemia and a weak Th1 immunological response is likely the main physiological mechanism that leads to progressive PKDL.

**Conclusions:**

A combination of 14 biochemical variables identified by unsupervised ML was able to detect a worsening PKDL state in Sudanese patients. This approach could prove instrumental to train future supervised algorithms based on larger patient cohorts both for a more precise diagnosis and to gain insight into fundamental aspects of this complication of visceral leishmaniasis.

## Author summary

Post-kala-azar dermal leishmaniasis (PKDL) is a neglected disease that causes stigmatization and carries a significant socioeconomic and personal burden in South Asia and East Africa. Currently, predicting its progression to a severe form remains largely unknown. In this study, we used machine learning (ML) methods to identify biomarkers of disease progression in PKDL using clinical, biochemical, haematological, and immunological data from a large cohort of Sudanese patients with either progressive (worsening) or stable conditions at diagnosis. For the first time, this study identified a combination of patient factors that may help provide a more accurate diagnosis of difficult-to-treat PKDL cases, with potential implications for improving patient management and quality of life.

## Introduction

Post-kala-azar dermal leishmaniasis (PKDL) is a dermal complication of visceral leishmaniasis (VL) caused by *Leishmania donovani*. Presently, it emerges as a new neglected disease seen mainly in two regions: South Asia (India, Nepal, Bangladesh) and East Africa, where Sudan shows the highest incidence [1–4]. Although mortality from PKDL is low, it is a stigmatizing disease that carries a significant socioeconomic and personal burden [5,6]. There is also concern that patients with PKDL could be a reservoir of VL, and its eradication has been linked to effective VL control [1–4].

The clinical features of PKDL differ from South Asia and East Africa. In South Asia, PKDL manifests as a chronic dermal condition with polymorphic lesions (coexistence of macules/patches along with papulonodules) [7]. This form of PKDL develops in 5-10% of patients and usually appears 2-3 years after VL treatment [3,8]. In East Africa, particularly in Sudan, PKDL include a hypopigmented macular or erythematous maculopapular rash around the mouth (interfering with feeding in the very young when involving the oral mucosa) and trunk, which may gradually extend to the entire body. Nodular forms and mixed types of skin lesions are also observed. The typical distribution pattern has resulted in the description of three clinical grades of severity, grade 2 and 3 being the more severe. In Sudan, 50-60% of VL patients develop PKDL either after VL remission or even during treatment. PKDL lesions often self-heal after 12 months, with only more severe cases requiring treatment [3,8].

In Sudan, recommended treatment is a combination of sodium stibogluconate (SSG) 20 mg/kg plus paromomycin 11 mg/kg for 17 days or SSG 20 mg/kg/day for 40–60 days. These regimens carry a high risk of kidney and liver toxicity and require prolonged hospitalization and painful daily intramuscular injections [1]. If treatment is not satisfactory, it can be extended by another 1-2 months. As second line treatment, liposomal amphotericin B can be given in hospital at 2.5 mg/kg/day for 20 days. These shortcomings mean that treatment is only recommended if the disease is disfiguring (grade 3), lesions are progressive, it is concomitant with anterior uveitis, or if patients are young children with oral lesions that interfere with feeding [9,10].

Symptoms and disease course are heterogeneous, and disease status classification only relies on late clinical assessment. From the appearance of PKDL lesions, it takes at least 6 months to identify who will progress to a worsening phenotype. However, while there is a clear need to determine how to prioritize the early treatment of these cases, currently no measurable factors associated with a risk of progressive disease have been identified, primarily due to the limited understanding of the mechanisms involved in the progression of skin lesions in PKDL. It is hypothesized that inflammatory reactions and dysregulated inflammasome activity can lead to skin hypopigmentation and facilitate *Leishmania* persistence [11]. Additionally, chronic kidney disease can also cause skin issues [12,13] and renal involvement may occur due to VL-related kidney damage and nephrotoxic therapy [14]. But, as mentioned above, limited studies have been performed to explore the involvement of host immunological responses or the contribution of specific tissues to the PKDL development, likely because its neglected disease condition.

New techniques based on machine learning (ML) methods are proving to be efficient alternative tools for the diagnosis, stratification and treatment outcome prediction of infectious diseases [10]. ML facilitates the accurate classification of elements in complex problems based on selected feature subsets. Although ML has been successfully applied to bacterial and viral infections such as sepsis and COVID-19 [11,12], respectively, its use for rare or neglected tropical diseases like PKDL remains largely unexplored.

The aim of this study was to identify biomarkers of disease progression in PKDL. To this end, we used a prognostic machine learning approach based on clinical, biochemical, haematological and immunological data obtained in 110 Sudanese patients. The detection of specific factors associated with a worsening disease phenotype may help to rationally substantiate the diagnosis of difficult-to-treat PKDL cases for early preventive treatment.

## Methods

### Ethics statement

For the present study, we only analyzed variables determined in PKDL patients during the baseline examination following enrolment (before treatment) from an already published clinical trial (ClinicalTrials.gov NCT03399955). Safety, efficacy and immunological results of each arm were previously published in the context of the clinical trial (Torres et al, 2024; Younis BM et al., 2023 [2,15], Supporting information: S1 Trial Protocol and S1 CONSORT Checklist; ClinicalTrials.gov NCT03399955). Approval was granted by the independent ethics committee at the Faculty of Medicine, University of Khartoum, and the Sudanese National Medicines and Poisons Board (Ref. FM/DO/EC, Date 8.8.2019). The study adhered to the Declaration of Helsinki, the International Council for Harmonization Good Clinical Practice (GCP) guidelines, and all relevant state, local, and international laws protecting human subjects’ rights and welfare. Informed consent and assent (when applicable) were obtained in line with regulatory requirements. Written voluntary informed consent was secured from adult participants and from the parents or guardians of children under 18 years old; assent from minors was also obtained as per country regulations.

### Recruitment of patients

Our study was conducted in Geradef state, Sudan. Participants were patients with documented stable disease, disease progressing over at least 6 months, or grade 3 disease, as confirmed by clinical presentation and the detection of parasites either in a skin smear or by PCR [more details in [15]].

Patients whose disease state was described as progressive (or worsening) phenotype were those whose lesions were seen to worsen in 6 months. Those with lesions that remained unchanged over the same time period were described as having stable disease. These two clinical states were used to stratify patients and to identify associated haematological, biochemical and/or immunological factors.

Details of participant characteristics may be found in S1 Table

### Clinical examinations

#### Medical history.

The medical history of each patient was recorded including PKDL type (macular, papular, maculopapular or plaque-like), disease state (stable or worsening in the past 6 months), number of months of disease, and PKDL severity grade in terms of lesion distribution and density (grades 1-3) [16]. For lesion distribution grading: grade 1 = mainly on the face with some lesions on trunk and arms; grade 2 = face, upper parts of the trunk, arms, and legs (gradually becoming less distal), hands and feet free of lesions; and grade 3 = all over body, including hands and feet. Lesion density was graded as: grade 1 = scattered lesions; grade 2 = moderate density with normal skin between lesions; and grade 3 = dense rash, no normal skin [17,18]. Patients with grade 2 or 3 in terms of both lesion distribution and/or density are therefore considered to have severe disease. In addition, we recorded the VL treatment received: 60% of the patients had been given SSG and paromomycin, and 39.1% SSG [15].

#### Clinical laboratory tests.

Upon examination, 6 mL of blood were collected: 1 mL in EDTA tubes (haematological analysis), 2 mL in a dry tube (biochemical analysis) and 3 mL in lithium heparin tubes (cytokine assays) [15].

Ten haematological and 6 biochemical variables were determined: haematocrit, haemoglobin, RBC count, WBC count and differentials (lymphocytes, monocytes, neutrophils, basophils, eosinophils), platelet count; and plasma concentrations of albumin, creatinine, potassium, alanine aminotransferase (SGOT/ALT), aspartate aminotransferase (SGPT/AST), and bilirubin (S1 Table).

### Immunological status

#### Stimulation of whole blood with soluble Leishmania antigen (whole blood assay [WBA]).

Whole blood samples were stimulated as previously described [19,20]. Briefly, for each sample an aliquot of 500 μL of blood was transferred to a control tube (unstimulated), a tube containing 5 μg soluble *Leishmania* antigen (SLA) from *L. donovani*, and a positive control tube with 5 µg of phytohemagglutinin (PHA). The antigen preparation of *L.donovani* antigen (SLA) was carried out as previously described [21]. All tubes were then incubated at 37°C for 24 h. After incubation, supernatants were collected and stored at -80°C until cytokine/chemokine measurement.

#### Cytokine and chemokine determinations.

For immunodetection of cytokine and chemokine levels, a magnetic multiplex kit for 12 human cytokines (Human XL Cytokine Luminex Performance Base Kit, cat# LUXLM000), a high sensitivity kit for the determination of IL-22 and IL-23 (Human IL-22 High Sensitivity Magnetic Luminex Performance Assay; Human IL-23 High Sensitivity Magnetic Luminex Performance Assay, cat# LBHS5782 and LBHS1716) and a kit for TFG-β1 (TGF-beta 1 Magnetic Luminex Performance Assay, cat# LTGM100) were purchased from R&D Systems (Minneapolis, MN, USA) and used following the manufacturer’s instructions. The following 15 immunological factors: IFN-γ, IL-1β, IL-2, IL-4, IL-5, IL-13, IL-17A, IP-10, PDL-1, TNF, granzyme B, IL-10, IL-22, IL-23 and TFG-β1 were quantified as described elsewhere [20]. Data were acquired using a Bio-Plex 200 system (Bio-Rad, CA, USA) with automatic clustering and analyzed using Bio-Plex Manager Software (Bio-Rad).

Results for each chemokine/cytokine were expressed as the difference between SLA-stimulated and control plasma concentrations in pg/ml.

#### Statistical analysis.

Data analysis was performed with GraphPad Prism v9.0 software (GraphPad Software, USA). Normality was examined using the Shapiro-Wilk test, and the non-parametric Mann-Whitney U test (two-tailed test) used to analyse differences between unpaired groups. Significance was set at p ≤ 0.05.

### Statistical analysis and unsupervised machine learning

Numeric procedures were carried out using Python standard libraries and in-house scripts [22].

The normality of the distribution of each variable was determined by the Shapiro–Wilk test using the *scipy.stats.shapiro* function/module. The *scipy*.stats.ttest and scipy.stats.*mannwhitneyu* Python methods were used for the two-sided parametric Student’s t-test and non-parametric Mann-Whitney U test, respectively. The r value of linear regression was calculated with the *scipy.stats.linregress* module. Properties containing zero values for more than 80% patients were deemed sparse and not considered thereafter. Data were normalized using *sklearn.preprocessing.robustscaler*, a scaler that process data considering the median and the quantile range, and thus reduce the impact of outliers. Dimensionality was reduced through principal component analysis (PCA) using the *sklearn.decomposition.PCA* procedure. Clustering of unsupervised machine-learning was conducted by the K-means method using *sklearn.cluster.KMeans* with default values including Euclidian distances, except the k (number of cluster) parameter of the Scikit-learn suite [22]. K-mean screening was conducted with an in-house script programmed in Python. The significance of the relationship between cluster content and disease state at baseline was assessed by the two-sided Fisher’s exact test using the *scipy.stats.fisher_exact* module. For agglomerative hierarchical clustering, we used *sklearn.cluster.AgglomerativeClustering* and *scipy.cluster.hierarchy.dendrogram* methods. Data were visualized with the matplotlib library. For supervised learning, logistic regression with ten non-redundant relevant features was carried out using *sklearn.linear_model.LogisticRegression* and considering class weight. Grid hyperparameter tuning was performed done with the following values: (l1 and l2), (0.1, 1, 10) and (‘lbfgs’, ‘liblinear’,’saga’) for penalty, C and solver parameters, respectively. Assessment of model performance were carried out through Matthews correlation coefficient (MCC) using the *sklearn.metrics.matthews_corrcoef* method, due to class imbalance, and accuracy with *sklearn.metrics.accuracy_score* method. For that, data were divided into 50% training and testing with *sklearn.model_selection.train_test_split*, or the application of either five-fold cross-validation using *sklearn.model-selection.KFold* or leave one out protocol with *sklearn.model-selection.LeaveOneOut*.

## Results

### Cohort dataset and description of feature scheme

Twenty-eight of the PKDL patients (25%) satisfied clinical criteria for a worsening disease state, while the remaining individuals (82 patients, 75%) were considered to have stable PKDL. Lesion distributions and densities for these two phenotypes are provided in Table 1.

**Table 1:** Clinical variables according to a stable or worsening PKDL state.

	STABLE	WORSENING	TOTAL
**N** (%)	82 (74.54)	28 (25.45)	110
**Age (years),** median (IQR)	9 (7-11)	9 (7-10)	9 (7-10)
**PKDL type,** n (%)			
Macular	1 (1.22)	2 (7.14)	3 (2.73)
Maculopapular	65 (79.27)	20 (71.43)	85 (77.27)
Papular	14 (17.07)	6 (21.43)	20 (18.18)
**Distribution of lesions,** n (%)			
Grade 1	66 (80.49)	18 (64.29)	84 (76.36)
Grade 2	13 (15.85)	7 (25.00)	20 (18.18)
Grade 3	3 (3.66)	3 (10.71)	6 (5.45)
**Density of lesions**, n (%)			
Grade 1	55 (67.07)	16 (57.14)	71 (64.55)
Grade 2	22 (26.83)	11 (39.29)	33 (30.00)
Grade 3	5 (6.10)	1 (3.57)	6 (5.45)

**Fig 1:**
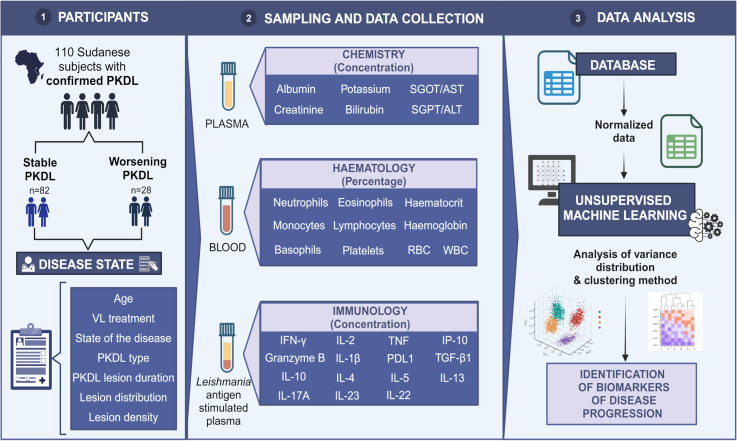
Physiological data framework. Patient metadata and feature scheme. Created in BioRender. Torres, A. (2025) https://BioRender.com/j75e448.

Thirty-one factors were determined: 6 biochemical, 10 haematological and 15 immunological (Fig 1).

### Conventional statistics and feature correlations

The discriminatory capacity of the 31 variables used to predict disease phenotype was assessed by conventional parametric and non-parametric statistical methods.

The predictive power of independent features was calculated for those with progressive (worsening) disease versus stable disease. Twenty-seven features followed a Gaussian distribution (p < 0.05, Shapiro–Wilk test) (S2 Table). The median values of all features analyzed fell within normality ranges, except for haemoglobin level in the worsening group (S1 Table). Only four of these properties (creatinine, potassium, total bilirubin and TNF) showed weak significant association with the worsening phenotype (0.05 < p > 0.01, Student’s t-test). None of the four non-normally distributed properties yielded significance as assessed by the Mann-Whitney U test (Table 2). The limited performance overall of the univariate approach suggested the use of multivariate procedures.

**Table 2:** Variable analysis using conventional statistics.

Variable	STABLE	WORSENING	Stable vs Worsening
	Mean±SD	Mean±SD	*P-value*
Haematology			
Basophils (%)	0.00 ± 0.00	0.00 ± 0.00	NC
Eosinophils (%)	2.33 ± 1.51	2.71 ± 1.82	0.272
Haematocrit (%)	37.11 ± 3.05	36.29 ± 4.65	0.287
Haemoglobin (g/dL)	12.54 ± 1.05	12.01 ± 1.71	0.057
Lymphocytes (%)	45.70 ± 8.05	45.14 ± 9.63	0.766
Monocytes (%)	7.74 ± 2.74	8.07 ± 3.14	0.600
Neutrophils (%)	44.23 ± 8.64	44.11 ± 9.79	0.949
Platelets (x10E^3^/µL)	347.99 ± 99.88	322.61 ± 79.50	0.226
RBC (x10E^6^/µL)	4.71 ± 0.40	4.58 ± 0.60	0.199
WBC (x10E^6^/µL)	7.34 ± 3.75	6.62 ± 1.91	0.334
Biochemistry			
Albumin (g/L)	39.07 ± 4.16	39.31 ± 4.79	0.799
Creatinine (mg/dL)	0.39 ± 0.20	0.49 ± 0.17	0.021
Potassium (mmol/L)	3.94 ± 0.33	4.12 ± 0.30	0.011
SGOT/AST (U/L)	30.99 ± 10.17	31.43 ± 9.73	0.842
SGPT/ALT (U/L)	27.11 ± 12.71	24.86 ± 11.39	0.408
Total bilirubin (mg/dL)	0.51 ± 0.29	0.37 ± 0.25	0.029
Immunology			
Granzyme B (pg/mL)	155.04 ± 307.11	41.23 ± 70.91	0.055
IFN-γ(pg/mL)	564.57 ± 826.46	287.55 ± 379.32	0.090
IL-1β(pg/mL)	457.07 ± 577.40	231.17 ± 326.78	0.052
IL-2 (pg/mL)	123.59 ± 204.16	53.17 ± 78.77	0.079
IL-4 (pg/mL)	0.26 ± 0.93	0.16 ± 0.59	0.603
IL-5 (pg/mL)	0.77 ± 1.70	0.49 ± 1.28	0.415
IL-10 (pg/mL)	39.66 ± 94.29	21.22 ± 58.14	0.333
IL-13 (pg/mL)	4.68 ± 13.03	4.57 ± 8.50	0.967
IL-17A (pg/mL)	7.02 ± 31.17	1.83 ± 4.68	0.384
IP-10 (pg/mL)	1555.06 ± 1140.01	1996.36 ± 1372.48	0.096
PDL-1 (pg/mL)	10.36 ± 19.05	8.28 ± 10.49	0.584
TNF (pg/mL)	612.65 ± 935.65	181.19 ± 318.34	0.019
IL-22 (pg/mL)	51.36 ± 126.20	15.07 ± 57.03	0.145
IL-23 (pg/mL)	0.12 ± 0.60	0.24 ± 0.93	0.425
TGF-β1 (pg/mL)	12216.23 ± 20683.62	7604.68 ± 11864.16	0.267

**Fig 2:**
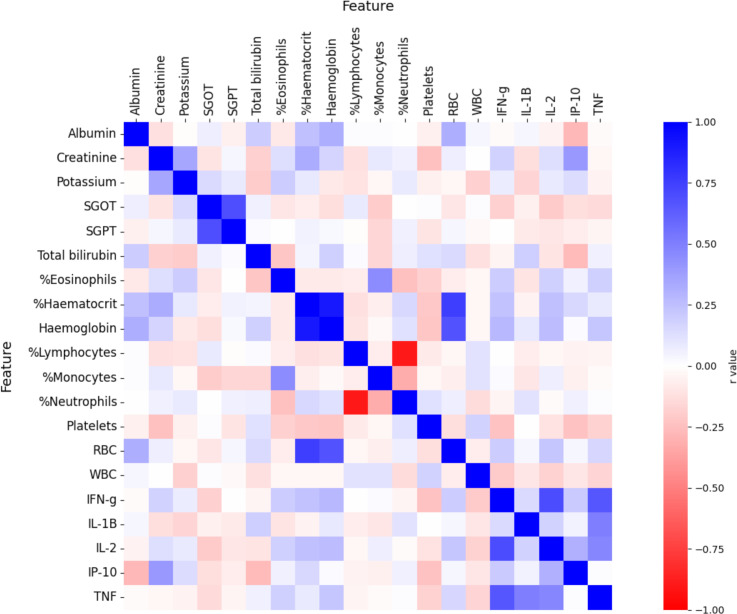
Heatmap indicating r values for all-against-all properties > 80% non-zero numbers. The chart was built with the *heatmap* function of the *seaborn* Python library.

Before testing the effects of any feature combinations, correlations between all variables were calculated (Fig 2). Expected dependencies were detected (indicated in dark blue in Fig 2) among haemoglobin, %haematocrit and RBC (r ≥ 0.68, p < 10^-15^), and between the liver enzymes SGOT and SGPT (r = 0.69, p = 5 x 10^-17^), supporting the accuracy of these experimental measurements. Levels of the pro-inflammatory interleukins IFN-γ, IL-2 and TNF (r = 0.47 – 0.70, p < 10^-6^) were also highly correlated. Further, when we examined ‘r’ values instead of ‘r^2^’, anti-correlations were also detected (indicated in red in Fig 2). The most remarkable example was %lymphocytes and %neutrophils that were anti-correlated with r value of -0.90 (p = 10^-40^). All in all, detecting expected correlations serves as technical quality control of the measurement analysis process.

### Unsupervised learning: PCA and clustering

In view of the limited univariate statistical performance of independent biomarkers, we adopted a multivariate analysis approach through ML. These techniques are able to find hidden multi-feature patterns underlying non-linear relationships in the data. Unfortunately, the use of supervised approximations in our case has several limitations. These include the small size of our cohort and class unbalance (only 25%, 28 worsening patients) involving insufficient “a priori” labelled data for training and validation, besides the uncertainty of whether PKDL corresponds to a unique pathophysiological mode. Instead, we explored the data through unsupervised ML techniques. This alternative strategy serves to detect natural groups of patients using the data in an agnostic way, and these groups can be analyzed later applying distinct classificatory scenarios [23,24].

For this purpose, it should be first noted that features show a wide range of magnitudes, sparsity, and the presence of outlying data. Despite their continuous nature, many measures were below the limits of detection (i.e.,: null). Thus, only 21 properties (of the 31 examined) showing non-zero values for >80% subjects were considered for further analysis (S2 Table). Notably, Th2 cytokine levels produced after SLA stimulation were close to zero, and these data were discarded during normalization for PCA and clustering. Next, we conducted a preliminary data normalization step using a scaling method based on medians and interquartile ranges to avoid the overinfluence of some factors and particular outliers.

**Fig 3:**
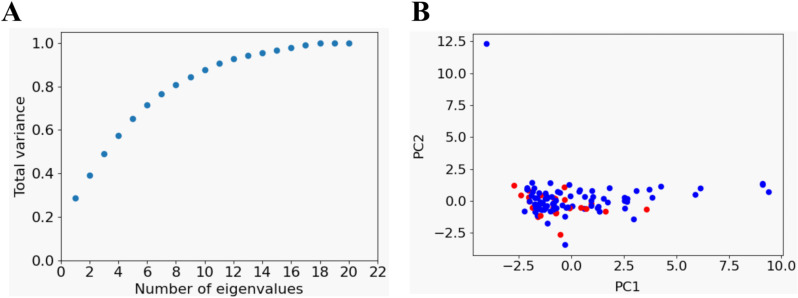
Dimensionality reduction by PCA. (A) Dimensionality reduction using robust normalized data. Cumulative variance by PCA. (B) Bidimensional scatter plot showing patient data for “stable” (blue) and “worsening” (red) PKDL.

In a second step, we reduced data dimensionality for feature selection by principal component analysis (PCA). Eleven principal components (PCs) accounted for > 90% (90.7%) of the total variance (Fig 3A). Ten properties were selected as the most important variables contributing to the eleventh PCs, as IL-2 was calculated as such for two of these PCs: albumin, potassium, SGPT, %haematocrit, %lymphocytes, platelets, WBC, IFN-γ, IL-1β and IL-2.

The two principal eigenvectors, despite explaining ≈39% of the total variance (27.8% and 11.3%, respectively), still showed an intermingled worsening versus stable pattern (Fig 3B). The fact that data from subjects with both phenotypes were dispersed across the graph indicates that disease state relies on a number of factors in our scheme whose variance exceeds that of the two features with the higher variance.

Finally, we considered that clustering methods would capture more complex correlations between variables and PKDL state using the non-redundant 10mer property subspace. For this, we used K-means, as a widely used technique that offers easy-to-interpret results. K-means does not find an ideal number of clusters but this parameter (the k value) must be provided beforehand, and the clustering output then assessed by external metrics.

**Fig 4:**
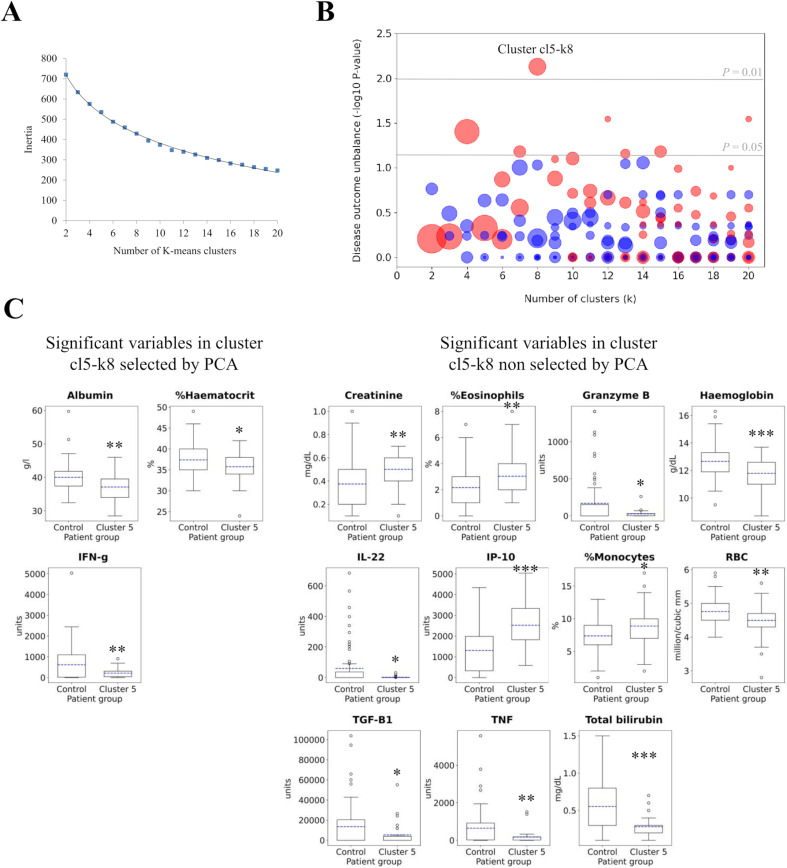
Clustering charts. (A) K-means clustering inertia for k values from 2 to 20. (B) Significance of clinical phenotype enrichment in patients with stable (blue) or worsening (red) PKDL disease for clusters with k values from 2 to 20. Sphere diameter is proportional to cluster size (number of patients). Dashed grey lines indicate significant (p < 0.05) and highly significant (p < 0.01) values for enrichment in either stable or worsening patients. (C) Boxplot panel showing value distributions of features, both PCA-selected and non-selected, significantly differing in cluster cl5-k8 compared to the remaining subjects. Boxes represent the interquartile range; the line is the mean. Whiskers indicate up to 1.5 fold the interquartile range. Outliers are shown in circles. Significance: *p < 0.05, **p < 0.01, ***p < 0.001.

K-means clustering was conducted with k values ranging from 2 to 20. The resulting clusters were assessed through consistency (or inertia, i.e., intracluster closeness) and associations with the worsening phenotype. Inertia decreased sharply from two to ten clusters; thereafter cluster partitioning did not remarkably change cluster compactness (Fig 4A). Significance for each cluster was calculated with *a posteriori* labelling in relation to the fraction of worsening patients to reveal if any of the clusters generated achieved relative enrichment of this phenotype with respect to the remaining clusters and absolute cluster size. Only two clusters showed a significant increase in the worsening phenotype being the most remarkable (p = 0.0074, Fisher’s exact test) the 5^th^ cluster at a clustering k value of 8 (termed here “cl5-k8”). This cluster contained 33 patients of whom 16 (48.5%) had a worsening disease state. This is 3.1-fold more abundant with respect to the remaining subjects (control) grouped in other clusters (12 worsening patients out of 77, 15.6%). This strongly suggests that the cl5-k8 cluster captures normalized values for the 10mer property subspace prone to the worsening behaviour (Fig 4B). Despite data scarcity prevent efficient supervised approaches, this was attempted with logistic regression and scaled data for the ten variables. Even after intensive hyperparameter tuning, maximal accuracies of 69% (MCC = 0.278), 61% (MCC = 0.252) and 66% (MCC = 0.278) were reached using 50% data for testing, five-fold cross-validation and leave-one-out validation methods, respectively. This modest achievement roughly concords with the fact that data unsupervisedly support a rather majority trend, cluster cl5-k8 here, but not the existence of a single and pure “worsening” class.

Additionally, our analysis of metadata from patients with severe PKDL (grades 2 and 3) within the cl5-k8 cluster identified lesion density as the only factor that showed a significant difference. Based on this association, patients were further stratified according to lesion density. Of the 33 patients in cluster cl5-k8, 26 patients (26/33, 78.78%) had grade 1 lesions whereas only 7 patients in this cluster (7/33, 21.2%) had a severe clinical condition (grades 2 and 3). In contrast, a larger proportion of patients with severe disease were found to group in another cluster: cl1-k8 (18/41, 43.9%).

When the original variable values for control and cl5-k8 subjects were recovered, these were within the normality range. However, they showed significant differences in terms of three factors used for clustering: albumin (40.0 ± 4.2 vs 37.1 ± 4.0 g/L, mean ± standard deviation, p = 0.0002, Student’s t-test), %haematocrit (37.4% ± 3.3 vs 35.8 ± 3.6, p = 0.025) and IFN-γ produced after SLA stimulation (615 ± 856 vs 211 ± 209 pg/mL, p = 0.009) (Table 3). Consequently, the other seven properties defining the PCA were not as relevant to describe a worsening condition.

**Table 3:** Cluster cl5-k8 values. Mean ± SD for original (non-normalized) PCA variables are shown with respect to the same ciphers for the remaining cluster subjects (control).

Feature class	Feature	Control	cl5-k8	P-value
Haematology	%Haematocrit	37.4 ± 3.3	35.8 ± 3.6	0.025*
	%Lymphocytes	45.3 ± 7.8	46.1 ± 9.6	0.61
	Platelets	348 ± 105	326 ± 64	0.25
	WBC	6.81 ± 1.64	7.97 ± 5.55	0.10
Chemistry	Albumin	40.0 ± 4.2	37.1 ± 4.0	0.0011**
	Potassium	3.96 ± 0.35	4.03 ± 0.27	0.41
	SGPT	27.6 ± 13.3	24.0 ± 9.3	0.16
Immunology	IFN-γ	615 ± 850	211 ± 206	0.0087**
	IL-1β	463 ± 590	251 ± 308	0.055
	IL-2	124 ± 210	63 ± 69	0.11

Significance: *p < 0.05, **p < 0.01

In addition, significant differences were found for 11 other features not considered for clustering after dimensionality reduction (Fig 4C and S2 Table). In combination with the three factors first identified, these also emerged as important to define the worsening phenotype.

These procedures identified a total of 14 variables able to properly define a worsening PKDL phenotype (Table 4).

**Table 4:** Variables defining worsening PKDL.

Variable	P-value cl5-k8 vs non-cl5-k8
Albumin (g/L)	0.0011
Creatinine (mg/dL)	0.00185
Total bilirubin (mg/dL)	0.0000014
Eosinophils (%)	0.00876
Haematocrit (%)	0.025
Haemoglobin (g/dL)	0.000686
Monocytes (%)	0.01
RBC (x10E6/µL)	0.00532
Granzyme B (pg/mL)	0.011
IFN-γ(pg/mL)	0.00871
IP-10 (pg/mL)	0.00000034
TNF (pg/mL)	0.00632
IL-22 (pg/mL)	0.015
TGF-β1 (pg/mL)	0.038

**Fig 5:**
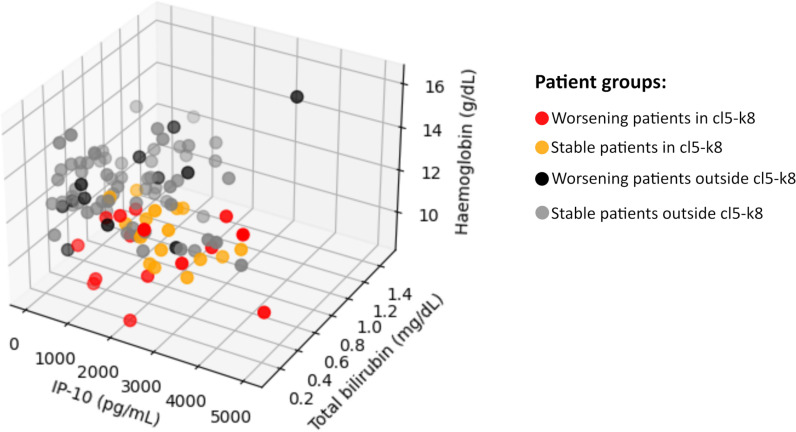
Spatial representation of three of fourteen features found to define the worsening PKDL phenotype. Red dots: worsening patients in cluster cl5-k8; Orange dots: stable patients in cluster cl5-k8; Black dots: worsening patients outside cluster cl5-k8; Grey dots: stable patients outside cluster cl5-k8.

When the three variables differing most significantly in cluster cl5-k8 were represented three-dimensionally, clear separation was observed between patients within and outside the cluster (Figs 5 and S1).

When compared to the remaining clusters, cl5-k8 showed relative enrichment in worsening subjects for both grade 1 (38.5% cl5-k8 vs 13.3% non-cl5-k8, p = 0.05, two-tailed Fisher’s exact test) and more severe disease, i.e., grades 2-3 (85.7% cl5-k8 vs 18.7% non-cl5-k8, p = 0.03). These results suggest that, even though grade 1 lesions are considered non-severe, the presence of the factors described within cl5-k8 may predispose these patients to disease progression.

In addition, several other trends were identified (S3 Table), namely a higher occurrence of subjects of younger age (8.9 ± 2.4 vs 9.8 ± 4.5 years, means ± SD) and a higher occurrence of the papular PKDL form (27.3% vs 11.3%) in cl5-k8. The 16 worsening patients in cl5-k8 were even younger on average (8.6 ± 1.9 years) and showed longer disease durations (34.3 ± 36.0 vs 26.6 ± 25.6 months). In none of these analyses, were other relevant factors, such as VL treatment, found to play a role in the worsening phenotype.

## Discussion

As the clinical manifestations of PKDL range from mild to severe, identifying specific patient factors associated with disease progression could help guide therapeutic decisions. In this study, single measurable factors were inefficient at predicting PKDL lesion severity, as only a modest relationship with PKDL worsening was found for four variables (creatinine, potassium, total bilirubin and TNF). In contrast, our ML study served to identify a combination of patient factors with potential implications for patient management.

Through filtered k-means clustering, several distinct clusters grouping clinical features and laboratory profiles were identified. One of these, the cl5-k8 cluster, comprised data from 48.5% patients with worsening phenotype, a proportion 3.1-fold higher than for the remaining patients in the cohort. Consequently, the features characterizing cl5-k8 seem to be a valuable set of biomarkers able to identify patients whose PKDL is progressing but who do not yet show severe lesions, as evidenced by the 78.78% of grade 1 patients within this cluster. In effect, only a small proportion of patients (21.2%) had lesion densities graded as 2 or 3. These PKDL patients had experienced progressive disease in the past yet were now stable within their severe disease state. The values of three factors (albumin, haematocrit and SLA-IFN-γ) selected for clustering differed significantly in the PKDL-enriched cluster compared to values in the outgroup individuals, indicating these features represent the differential physiological core of this cluster. Consequently, lower values of albumin levels, %haematocrit and SLA-IFN-γ production were found here to define progressive PKDL. When original dimensionality was re-established, a further 11 modified variables were detected in the main cluster. This meant that a worsening PKDL phenotype was also defined by in general lower levels of bilirubin, haemoglobin, and RBC, and of SLA-granzyme B, TNF, IL-22 and TGF-β production, and higher levels of creatinine, eosinophils, monocytes and SLA- IP-10 production. Despite several clinical meaning parameters were commonly detected in these patients, causal and precise combinations of value ranges for these properties would require further prospective studies. Otherwise, overinterpretation of connections between properties should be avoided with present data to prevent falling into the multiple comparison problem.

Through ML analysis of existing experimental data, we were able to identify key molecular players affecting the pathogenesis and severity of PKDL. We noted that PKDL patients showing a worsening condition had lower plasma bilirubin and albumin levels than those with stable disease, while liver enzymes were unaltered. In studies addressing PKDL, lower bilirubin levels were detected in Sudanese patients compared to healthy subjects [25]. Unfortunately, these variables have not been examined in PKLD patients classified according to disease severity. In patients with VL, higher AST, ALT and total bilirubin have been associated with abnormal liver function [26]. Hence, the only subtle biochemical alterations observed in subjects with PKDL could indicate the absence of noticeable hepatocellular injury [1,27], regardless of the density grade of their lesions. It has been reported that hypoalbuminemia impairs the healing of skin and mucous membrane lesions in patients with tegumentary leishmaniasis or cutaneous leishmaniasis [28,29]. In turn, this protein depletion inhibits fibroblast proliferation, lengthens the inflammatory period, reduces collagen synthesis and wound tensile force, limits the phagocytic capacity of leukocytes, and increases parasite survival [30]. Consequently, the lower plasma albumin observed here in individuals with progressive disease might be linked to worsening of their cutaneous lesions, although other possible causes include inflammatory states [31] or deteriorating kidney function [32–34]. In effect, we detected higher creatinine and potassium levels in the progressing PKDL-enriched cluster and in worsening patients of the whole cohort, respectively. These findings might be related to potential tubulointerstitial alterations arising during previous VL [35,36], although glomerular filtration is unaffected in PKDL [25]. Unfortunately, the literature lacks reports of hepatorenal alterations in Sudanese PKDL patients or mice models to improve our understanding of the impacts and of this disease and underlying causes of its progression.

The progressing PKDL-enriched cluster identified here included lowered indices of haemoglobin, red blood cells and haematocrit, with haemoglobin levels below the normality range. These blood variables have been described as unaffected in PKDL patients from India and Bangladesh [37], yet there are no records for Sudanese patients. Sudanese VL and PKDL patients have been described to feature lower haemoglobin and RBC levels [38–40]. Peculiarly, anaemia is a characteristic feature of patients with VL, regardless of whether they progress or not to PKDL [41]. Haemophagocytosis plays an important role in anaemia and has been related to hyper-activation of parasitized macrophages [42,43], mainly of heavily infected macrophages [43]. Given that patients with PKDL no longer show the leukopenia and anaemia seen in VL, the pathology of PKDL seems to be restricted to lesion site [37]. In our study, the abnormal haematological profile found in worsening PKDL patients suggests the dissemination of active infected macrophages throughout the skin, enhancing damage. Other factors like nutritional iron, folate or vitamin B12 deficiency could also cause these alterations [44].

Several immunological and molecular features were observed in our worsening cohort suggesting these are ineffective at controlling the infection. Increased monocyte and eosinophil counts have been reported in Sudanese patients with progressive PKDL, and this has also been described in Indian PKDL patients [45]. Some studies have shown evidence of eosinophil phagocytosis of the *Leishmania* parasite [46,47]. Eosinophils can shape the local and systemic response to leishmaniasis through the secretion of immunomodulatory cytokines [48]. Accordingly, eosinophils from subjects with VL have been found to downregulate Th1 IL-12 and IFN-γ cytokines in response to SLA [49]. In subjects with PKDL, monocyte/macrophage subsets appear to be alternatively activated (M2) to release anti-inflammatory cytokines and thus ensure *Leishmania* parasite survival and lesion chronicity [45]. In our patients with worsening lesions, SLA-specific IL-10 and TNF production was drastically diminished, and SLA-IP-10 levels increased. Although cytokines from monocytes were not measured, this profile might be related to M2 polarization [50]. In addition, we observed the low production of SLA-specific IFN-γ and granzyme B, indicating a poor Th1 response and debilitated NK and CD8^+^ lymphocyte cytotoxic response against *Leishmania*-infected cells. Further, low SLA-IL-22 levels were found in patients with progressive PKDL. IL-22 protects against tissue damage during cutaneous leishmaniasis [51,52]. This cytokine is secreted by Th17 cells when triggered by *L. donovani* antigens [53]. As widely known, the Th17 response complements Th1 when reducing the parasite burden in VL [54], inducing the local and systemic production of TNF and nitric oxide [4]. Consequently, a poor Th1 plus Th17 response is linked to a lack of immunological control, increasing the risk of leishmaniasis progression [53].

While the induction of IFN-γ, TNF, IL-2 and granzyme B by SLA has been widely linked to the successful resolution of VL [21,55–56], PKDL elicits a mixed Th1/Th2 immune response [41,57]. Although the immunological profile of progressive PKDL has not yet been described, based on our findings, a weaker Th1 response in these patients could be perhaps explained by anergy/exhaustion of T lymphocytes, and/or the low presence of memory cells or short-term memory against *Leishmania* [58]. This scenario would be compatible with the presence of the parasite beyond the skin in PKDL patients [3,40,59] and with a greater propensity to develop extended disease [60,61].

Our study is limited by the lack of literature reports providing biochemical, haematological and immunological data for individuals with progressing PKDL. This could be the consequence of the limitations of conventional statistics. Also, addressing rare endemic diseases using data-intensive tools is challenging due to difficulties in recruiting high patient numbers. The biomarkers detected in our constrained observational cohort may therefore not be extrapolatable to other endemic areas and should be validated. For example, the trends observed here of a younger age, longer PKDL duration, and papular PKDL type related to a worsening PKDL state require confirmation in studies based on metadata. Our multivariate analysis is a pioneer approach that needs to be completed with data from larger studies in PKDL patients from Sudan and from other countries such as India where a different phenotype and idiosyncrasy of PKDL prevails. Comparable feature schemes emerging from PKDL patient populations may be adequate to train supervised ML algorithms able to generate predictive tools for a precise and early diagnosis of PKDL. Importantly, these tools will help improve our understanding of this disease.

In conclusion, using conventional laboratory tests and advanced machine learning techniques we were able to identify specific variations in key biochemical, haematological and immunological factors related to the differential progression of PKDL. This study can be considered a first step towards predicting a worse PKLD state in patients following the resolution of VL. Further research is needed to determine the applicability in clinical practice of the biomarkers identified in this study.

## Supporting information

S1 Fig 2D representation comparing three features identified as defining the worsening PKDL phenotype.
(TIF)

S1 Table List of all the variables and data used in the study.
(XLSX)

S2 Table Statistical analyses performed on the 21 properties that were considered in stable vs. worsening patients and cl5 cluster vs. the rest of the clusters.
(XLSX)

S3 Table Statistics of the metadata comparison between cluster cl5 and the other clusters.
(XLSX)
