# Supplementary figures and images for: Unsupervised machine learning identifies biomarkers of disease progression in post-kala-azar dermal leishmaniasis in Sudan
Source: PLoS Negl Trop Dis. 2025 Mar 11;19(3):e0012924. doi: 10.1371/journal.pntd.0012924 (PMC11932480; doi:10.1371/journal.pntd.0012924)

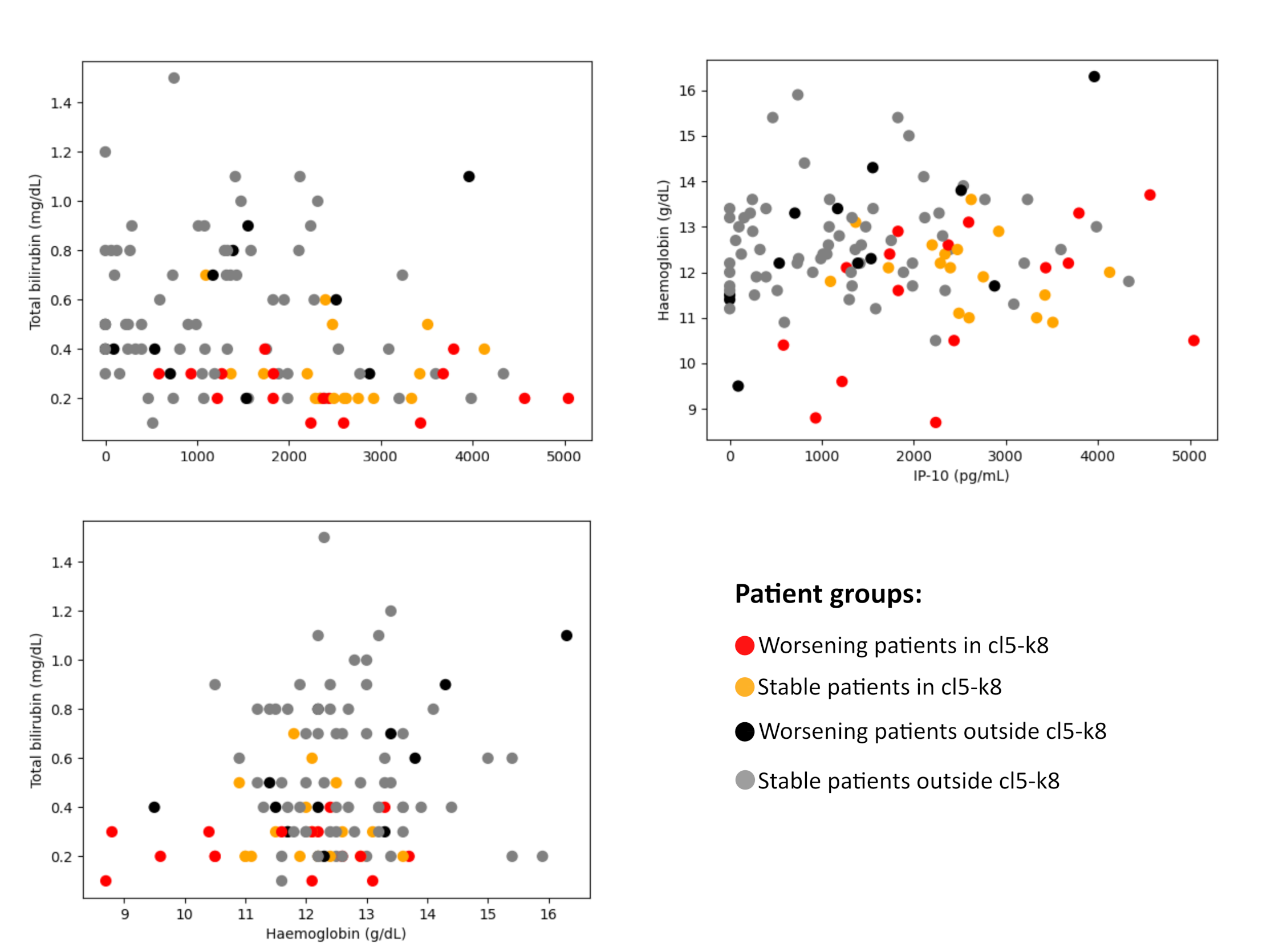

Supplement: S1 Fig — (TIF) [file pntd.0012924.s001.tif]
